# “Sacred Work”: Reflections on the Professional and Personal Impact of an Interdisciplinary Palliative Oncology Clinical Experience by Social Work Learners

**DOI:** 10.3390/geriatrics3010006

**Published:** 2018-02-03

**Authors:** Alyssa A. Middleton, Tara J. Schapmire, Barbara Head

**Affiliations:** 1 Kent School of Social Work, University of Louisville, Louisville, KY 40202, USA; alyssa.middleton@louisville.edu; 2 School of Medicine, University of Louisville, Louisville, KY 40202, USA; barbara.head@louisville.edu

**Keywords:** cancer, palliative care, older adults, interprofessional education, interdisciplinary teams, hospice, social work, learner reflections, experiential learning

## Abstract

This study explored the impact of an oncology palliative care clinical experience with older adults on social work learners. A three-member research team conducted a qualitative content analysis of reflective writings. 27 Master of Science in Social Work students enrolled in an interprofessional palliative oncology curriculum and completed a reflective writing assignment to summarize the clinical scenario, analyze the patient/family care provided, and describe the impact of the experience. Using a constant comparison approach based on grounded theory, the research team analyzed the reflections to come to consensus related to the overall impact of the experience. Two overarching themes (professional and personal impact) and 11 subthemes (appreciation of interdisciplinary teams, recognition of clinical skills of other disciplines, insight into clinical skills of the social worker, perception of palliative care, embracing palliative care principles, centrality of communication, importance of social support, family as the unit of care, countertransference, conflict between personal values and patient/family values, and emotional reactions) were identified. Experiential learning opportunities for social work learners in interprofessional palliative care build appreciation for and skills in applying palliative care principles including teamwork, symptom control, and advanced care planning along with a commitment to embrace these principles in future practice.

## 1. Introduction

As a profession founded on a strengths-based, patient-centered perspective, social workers are well-positioned to serve on palliative care teams and provide psychosocial support to patients with serious illnesses and their loved ones. This work, however, requires preparation and experience. Oncology, palliative, and hospice social workers must be trained and skilled in a variety of competencies including assessment, treatment planning, communication, interdisciplinary teamwork, pain and symptom management, and grief and bereavement care [1,2,3,4]. 

Training on the aforementioned competencies should begin in the classroom and extend throughout the career trajectory, with numerous options for hands-on learning and post-graduate continuing education [3,5,6,7]. However, this is often not the case. Despite calls for increased and more comprehensive education [3,5,7], little training exists in BSW (Bachelor of Social Work) or MSW (Master of Social Work)/MSSW (Master of Science in Social Work) programs for practice in palliative and end of life care [8,9,10,11].

Learners and practitioners report feeling anxious about death and dying and unprepared for tasks related to their positions and seldom receive preparation for practice within interdisciplinary teams [7,8,12,13,14,15,16,17]. As a result, interdisciplinary curricula have been developed to increase the skills and confidence of social work learners and other health disciplines to provide palliative care and/or hospice services to patients and families [15,18,19,20]. Evaluations from these interdisciplinary offerings—providing direct interactions with patients, families and other disciplines—demonstrate growth in both the professional and personal lives of learners [15,16,20,21,22]. Further review of social work learners’ perspectives is necessary to ensure interdisciplinary curricula is indeed incorporating social work tenets into the courses and training learners and future practitioners in best practices. Learners must develop skills not only to communicate and work with patients and families but also to advocate for their patients within the interdisciplinary team. Their ability to function on the team may be constrained by a history of hierarchy and turf wars [12,22]. Gaining feedback from learners about their learning experiences will help to strengthen and develop future training opportunities. 

At the University of Louisville, MSSW students interested in oncology/palliative care or geriatrics enrolled in the Interdisciplinary Curriculum for Palliative Oncology Care (iCOPE) [19,20] that included didactic online modules, a clinical experience, critical reflective writing and interdisciplinary group discussion about the reflective writings. The clinical experience took place in a variety of settings including hospitals and an inpatient hospice unit, and learners shadowed experienced social workers and observed cases involving middle to older aged adults. The purpose of this study was to explore the impact of a palliative care clinical experience with older adults on social work learners through qualitative analysis of their reflective writings. 

## 2. Materials and Methods 

Twenty-seven master’s level social work students completed a palliative care clinical experience in a setting that practiced team-based palliative care between fall semester 2013 and spring semester 2016. As part of participation in the iCOPE curriculum, students wrote a critical reflection paper on one of the older adult patients observed during their clinical experience. Students were required to complete the reflection. These 27 reflections were the data used in our analysis.

Qualitative data analysis was completed using a grounded-theory (GT) approach [23], meaning that the ultimate goal was not to generate a grounded theory, but rather to employ GT techniques throughout our coding and data analysis process. In accordance with GT, we used inductive coding, memo writing to document analytic decisions, and integrated ideas and concepts without allowing any one of them to drive or constrain our findings [24]. Constant comparative analysis was used to examine contrasts among respondents, situations, and settings. Data saturation was considered to have been achieved when the same themes reoccurred, and no new insights evolved from further data analysis. 

### 2.1. Description of Assignment

The critical reflection assignment (see Supplementary material for the full assignment description) consisted of three sections:

1. A brief summary of the clinical scenario
2. Critical analysis of the patient/family care provided, focusing on the interdisciplinary team approach and practice
3. Description/prediction of the professional and personal impact of the experience.

Students submitted their reflections electronically. Identifying information including learner and patient names was removed prior to analysis. Approval to use the writings for research purposes was granted by the University’s Human Subject Protection Program (study approval 12.0415) prior to any study activities. 

### 2.2. Methodological Approach

Each of the reflections was analyzed individually by each member of the research team, which consisted of a doctoral student with no affiliation to the iCOPE curriculum, and two iCOPE faculty members, neither of which were supervisors in the palliative care clinical experience. Analysis consisted of a “naïve” first reading of the first ten essays to acquaint the researcher with the data followed by a second reading in which data was read line-by-line and themes were coded using margin notations or the comment function in Word. The research team then met to develop an exhaustive list of themes identified. Consensus on the themes and subthemes was reached through peer debriefing and grouping of similar codes together. The initial codebook was created. 

An additional 17 reflections were reviewed in the same manner. At this point, no new themes emerged, and no deviant themes were identified. Team members used their analysis to select data (direct quotes) which were copied/pasted into groupings by subthemes. Team members reviewed the data together, summarized the overall findings and selected exemplary quotes within each subtheme. The full team developed consensus about the overall scheme of the data and interpretation of the findings. 

### 2.3. Ensuring Trustworthiness

Each member of the research team read all of the reflections and reviewed the analysis of other team members. Consensus regarding the coding was reached via dialogue and peer debriefing, attesting to the reliability of the overall findings. 

The process of conceptualizing themes, agreeing on subthemes, and developing an overall scheme was documented at each meeting. Therefore, a clear audit trail was ensured, and the approach could be replicated.

There were several threats to trustworthiness, one being respondent bias. Because the students wrote these reflections as a required component of the curriculum, they may not have been totally truthful or may have written the paper in an effort to gain faculty approval. Because two members of the research team were involved in the creation and implementation of the iCOPE curriculum and were invested in its success, researcher bias is also a possible threat to trustworthiness. This was offset somewhat by having a third member of the research team who had no relationship to, nor history with the project. 

## 3. Results

Eleven subthemes under the two major themes of professional and personal impact were identified. 

### 3.1. Professional Impact

What follows is a summary of the findings related to each of the subthemes within the two themes. Following each summary are Table 1, Table 2, Table 3, Table 4, Table 5, Table 6, Table 7, Table 8, Table 9, Table 10 and Table 11 reflecting exemplary quotes for each of the subthemes. 

#### 3.1.1. Appreciation of Interdisciplinary Teams

Learners often wrote about their observations of the teams and teamwork. As one learner said, it was “eye-opening” to actually observe how team members worked together in the care of a patient. Characteristics of teams noted by the learners included: cohesiveness, functionality, respect for each other and the patient, timeliness, trust, warmth, and compassion. Each team member was observed as having their own individual responsibility according to his/her scope of practice while also understanding the roles and contributions of fellow team members. The importance of both individual and team effort was noted by several students. 

#### 3.1.2. Recognition of Clinical Skills of Other Team Members/Disciplines

Team members observed by the learners included physicians, pharmacists, nurses, financial counselors, chaplains, psychologist, dietitians and social workers. For the learners, it was often the first time they observed these professionals in palliative care roles. The expertise of physicians and nurses in pain and symptom management was cited. Nurses were viewed as having the most knowledge about the physical care of the patient. The pharmacist’s role in assessing and managing medications and their side effects was an area of pharmacy practice not previously understood by some. Several learners were impressed by the communication and assessment skills of the psychologist. Both social workers and the psychologists were observed assisting with non-pharmacological symptom management. 

#### 3.1.3. Insight into Social Worker Clinical Skills

As these were social work learners, the data were rich with discussions of what learners appreciated about the role of the social worker. Insights included how well the social worker communicated with patients, families, and colleagues, how assessment questions came across as casual, yet supportive and relational conversation, and how much the social workers improved the emotional well-being of those with whom they worked, including both patients and the patients’ adult children. 

#### 3.1.4. Perception of Palliative Care

Learners were mixed in their reports on the impact of the experience on their perception of palliative care. Many reported an improved or enhanced perception, while others reported that their experience did not necessarily change their perception of palliative care, but rather strengthened their positive perception and reaffirmed their opinion that palliative care was important and essential for seriously ill patients and their families. Several commented on the need to offer palliative care earlier in the disease trajectory. Learners also expressed the importance of education—with patients, families and other health care professionals—to explain the nuances and benefits of palliative care, and how it differs from hospice care. 

#### 3.1.5. Embracing Principles of Palliative Care 

Several of the learners noted that the experience either affirmed or ignited their desire to work in this social work specialty area. While not all of these learners planned to practice in palliative care, all wrote about how they planned to apply what they learned about the principles of palliative care to their future practice including embracing the family as the unit of care and treating more than just the physical, to communicating with more compassion. They also discussed a commitment to introduce palliative care sooner for all seriously ill individuals. 

#### 3.1.6. Centrality of Communication

During their clinical experience, learners observed important communication skills including: use of open-ended questions; accepting silence; immediacy of team communication; developing rapport; intra and extra team communication; team rounds; importance of family meetings with the team; and orchestrating difficult conversations. In many cases, learners also noted communication problems such as the team not meeting together with the patient, the lack of communication between physicians and other team members, delayed communication and information sharing with the patient and family, and poor communication with other teams or providers (besides the palliative or hospice team) caring for the patient.

#### 3.1.7. Importance of Social Support

The presence or lack of adequate social support was often noted as an important consideration when working with seriously ill and dying patients. Having greater social support from family, friends and the team was viewed as critical to improving the patient’s quality of life. Patients without social support were seen as having more need of social work services as a means of providing adequate support.

#### 3.1.8. The Family as the Unit of Care

Learners were able to observe and put into practice the core social work value of considering the person in their environment, in that the palliative team focuses on the family and not just the cancer patient as the unit of care. This was reinforced repeatedly in the observations. 

### 3.2. Personal Impact

What follows are summaries of the findings related to each of the subthemes within the personal impact theme. Following each summary are tables reflecting exemplary quotes for each of the subthemes. 

#### 3.2.1. Countertransference

Countertransference is generally understood in social work to be the therapist’s emotional entanglement with a client. Many learners wrote about the personal challenges they faced in working with palliative care patients and the intensity of their feelings towards patients and family members. They recognized that countertransference was an important issue and shared several examples of this occurring in practice. 

#### 3.2.2. Emotional Reactions

The clinical experience generated a variety of emotional reactions. Learners described their reactions in a variety of ways including being touched, being overcome by emotions, feeling sad and heartbroken, being truly moved, being inspired, expressing feelings of fright and feeling good and fulfilled. Several commented that this was the first time they had experienced such strong emotion or observed such touching interactions between patients and their families or team members. 

#### 3.2.3. Conflict between Learner Values and Patient/Family Values

Social work learners and practitioners frequently encounter situations where personal values conflict with those of a client or family. This experience was no exception and provided an important opportunity for self-reflection and awareness. 

## 4. Discussion

In this study, we evaluated the professional and personal impact of an oncology palliative care experience with older adults on social work learners via evaluation of their reflective writings. Most learners reported a positive oncology palliative care experience and a number identified growth in their understanding and appreciation of the critical skills demonstrated by members of the interdisciplinary team including the team social worker. It is important to note that learners had varying experience with and exposure to palliative care before placement, so preexisting perceptions of palliative care may have been enhanced, rather than developed solely from this experience. Also, only students with an interest in oncology/palliative care or geriatrics were enrolled in the curriculum, which may have also biased the primarily positive experiences. 

Results were consistent with findings and recommendations by leaders in the field, including Reith and Payne [4] and Supiano and Berry [15] that experiential learning environments and opportunities for reflection help to develop skills necessary for social work learners to effectively participate on interprofessional teams These learner reflections also highlighted how social work competencies identified by Gwyther et al. [2] including assessment, interdisciplinary teamwork, and practice values such as sensitivity, examination of one’s own values about death, and willingness to work with others to provide care are developed and strengthened as a result of participating in an interprofessional curriculum.

As with other studies of interprofessional curricula, our study demonstrates that interprofessional, experiential learning can lead to increased growth in both the professional and personal lives of learners [15,16,20,21,22]. This type of experience can better prepare social work learners for the real-world scenarios they will face after graduation and begin building skills necessary for working with an interdisciplinary team. Better preparation would include more interprofessional experiences, participating in experiential learning activities, having mentors whom are experienced in palliative care and having facilitators who are well-versed in interprofessional teaching instead of historically didactic training. These types of preparation can create a larger toolbox of strategies and approaches from which social workers can draw as they work with patients and families.

The strengths of this study include the trustworthiness established by independent review of reflective writings by each team member, consensus on themes and subthemes and documentation of a clear audit trail. Limitations within this study merit discussion and consideration when interpreting findings. We were not able to use prolonged engagement with our study participants as the data were drawn from a reflective writing assignment and learners were de-identified. Triangulation of multiple sources of data was also not possible. Our sample was somewhat small and limited to students from one school of social work, though we did analyze to data saturation. Clinical experiences occurred in palliative care settings in one metropolitan area. Respondent and researcher bias (discussed in the earlier section on trustworthiness) were also potential threats to the validity of our findings. 

### Implications for Psychosocial Education and Practice

As social work learners will, at some point in their careers, interface at some level with clients or family members facing a serious illness and work with interprofessional teams, it is imperative that learners are exposed to palliative care principles and given the opportunity to develop and apply practice skills in this area. Implications from this study for psychosocial education and practice include:

- Learners—when exposed to palliative care principles and experiential learning opportunities in this field—express commitment to applying these principles in their future practice, regardless of their area of specialty. Programs such as iCOPE should be replicated and expanded in order to further the skills of social work learners.
- Providing opportunities for social work learners to observe and interact with interprofessional teams solidifies the importance of both individual and team effort on an interprofessional team for the learner.
- Clinical experiences such as these offer the opportunity for learners to observe seasoned social workers in real time, real-world environments. Learners can then identify positive and effective means of communication surrounding difficult topics that appear to be informal and conversational yet gather critical information to complete an assessment. Having experienced palliative care social workers as role models can improve learners’ communication skills and increase the comfort level and ease of social workers in training.

## 5. Conclusions

This study supports the inclusion of experiential, interprofessional geriatric, oncology and palliative care curricula within social work programs to better prepare social work learners for their futures in practice environments. These environments are increasingly requiring interdisciplinary teamwork and the ability to face death and dying while supporting clients and families.

## Figures and Tables

**Table 1:** Quotes related to the subtheme of appreciation of interdisciplinary teams.

Subtheme	Quotations
**Appreciation of Interdisciplinary Teams**	- Characteristics I have learned and witnessed of high-quality palliative care teams include: communication, cohesion, knowledge, advocacy, support, and involvement of the patient and/or family.
	- I was strongly affected by the functionality of the palliative care team.
	- It was very pleasant to see an interdisciplinary team that worked together very well and treated each very amicably and very respectfully. Both social workers I shadowed spoke highly of their interdisciplinary team members and expressed a great deal of trust in the capabilities of every team member. This was possibly the biggest positive I noticed during my observation.
	- Having participated this palliative care meeting, I see the benefits of an interdisciplinary team. I watched the family ask questions and the team members defer to one another for answers. I believe the family was relieved to know that a team of qualified people were collectively working to see that their father received the care he deserves.
	- One positive effect I really enjoyed seeing in the palliative care team is the high autonomy they try to preserve in the patient.
	- Each team member was aware of the others’ responsibilities and worked together to give the family the best insight into current status and future care of the patient.
	- Working together and sharing responsibility, the team provided support and expertise for each other and the patient.

**Table 2:** Quotes related to the subtheme of recognition of clinical skills of other disciplines.

Subtheme	Quotations
**Recognition of Clinical Skills of Other Disciplines**	- The dietitian is utilized greatly due to the side effects that the patient has experienced such as lack of appetite.
	- The palliative doctor is uniquely qualified to help with pain management and treatment side effects while patients are going through treatments.
	- The psychologist went over some breathing techniques with the patient that can help him when he starts feeling anxious. The psychologist also talked with the family about the patient’s prognosis and how the cancer has spread and there was nothing the doctors could do to treat his cancer anymore.
	- The pharmacist talked about the various drugs that the patient could be put on that would not have severe side effects.
	- During rounds, the nurses would inform us how T was doing, going more in depth than the doctor.
	- I was impressed by how the palliative care physician and psychologist stayed with Mr. O until his pain and restlessness were under control. While the team physician worked on figuring out the right combination of medicines to ease Mr. O’s pain and restlessness, the psychologist provided soothing imagery and touch to distract Mr. O from the discomfort he was experiencing. By taking such care of Mr. O, his [adult] son’s distress was relieved and he was able to spend quality time with his dad during his final days and hours. This is a gift, given the number of people who die in pain.
	- Individually, the team assessed and continually re-assessed the patient in their specific areas of discipline.
	- The chaplain met with the family to address spiritual needs. The chaplain is also a marriage and family therapist so she facilitated several family sessions for the B family.

**Table 3:** Quotes related to the subtheme of insight into social worker clinical skills.

Subtheme	Quotations
**Insight into Social Worker Clinical Skills**	- The social worker explained the meaning of palliative care, options of support and treatment, options for discharge, evaluated the psychological status and offered support and conversations. The social worker also explained her job as an advocate for the patient and organized and led a family conference with the parents and the team. The patient was also supported to create a living will, as well as a healthcare proxy (power of attorney).
	- The social worker helped to reduce feelings of anxiety and hopelessness during her initial visit. The couple was very appreciative of the social worker’s help and admitted that they felt slightly better after being able to verbalize their fears and concerns.
	- I learned a lot of the painful questions that you have to ask as a social worker to be able to provide the best care for the patient. Some of these can be awkward and challenging, but the way the oncology social worker presented them was inspiring. She is very competent in her field of practice.
	- It was so wonderful to observe a seasoned social worker and how she interacts with the patient and family asking questions in a very casual way so as to obtain the necessary assessment information with them being none the wiser.
	- My experience with this patient was very overwhelming. I have never worked with a patient that was so vocal about their suicidal ideations and had a plan of how they would commit suicide. I learned a lot about how the tone of one’s voice affects the patient. The social worker was very calm and did not seem shocked by the patient but instead tried to understand the patient’s ideations.
	- The social workers’ focus was meeting the client where he was. She asked about depression, thoughts of suicide and family issues. The social worker discussed support, and clients concerns and wants. She asked the client about ways of coping. The patient responded by talking about how he uses distractions to cope with pain. He also talked about his love for reading books, and how he uses them to stay focused. The social worker focused on more continuous care and symptom control for the client.

**Table 4:** Quotes related to the subtheme of perception of palliative care.

Subtheme	Quotations
**Perception of Palliative Care**	- I was never sure of what palliative care was until I started working at the VA hospital and hearing about it in my psychosocial oncology class. At first, I thought it would be impossible because palliative care involves so many busy people that most wouldn't think it’s feasible for everyone to work together on one patient's care. After learning about the palliative care, I realized how impactful it could be in someone’s care and will now be a strong advocate for it. Once I saw it in action I now know how important palliative care is to someone’s care and if it can improve their quality of life it should be implemented in all forms of care.
	- I have learned the value of palliative care even more. There is a lot of support possible at the end of curative treatment and the interdisciplinary support from each team member. Palliative care does not mean to only reduce pain, but to give the day more life instead of the life more days.
	- This type of care helps patients and families have more control over their care, which leads to less anxiety and stress. Palliative care helps patients and their families carry on with their daily lives. Palliative care helps patient’s ability to go through treatment, which in many cases can lead to better outcomes. With the benefits that come with palliative care, it is also puzzling that it is not taken advantage of more often.
	- I think that the palliative team takes a more holistic approach in caring for patients who are not dying and in need of symptom and pain management. Because of the holistic approach taken by the team, I think more patients could benefit from their services earlier in the disease process.
	- Overall, the experience demonstrated how important palliative care and patient-oriented goal setting is for cancer patients and their families. The support and comfort palliative care brings should be an essential part of any cancer care agency.
	- I have realized that palliative care is essential at any stage of a patient’s journey. I see how important palliative care is for both patient and family members.

**Table 5:** Quotes related to the subtheme of embracing principles of palliative care.

Subtheme	Quotations
**Embracing Principles of Palliative Care**	- This experience touched me because it reinforced my passion for palliative care and my eagerness to be of service in helping families to navigate this portion of their journey. I look at people in this profession as guides and fellow travelers who are assisting people make their exit from this world while also helping their families as their loved ones’ transition. That might sound grandiose or romanticized but it is how I feel. This profession is all about “the important things in life”, relationships, love, dignity, compassion, fear and bravery, faith and so much more. I know that the day-to-day tasks of this profession aren’t always so lovely, but I believe at its essence, it is sacred work.
	- This experience with D and her family has motivated me to go above and beyond to really get to know patients and be an advocate for them, rather than going along with what may be the norm. Unfortunately, too many people I work with currently in my job are overlooked by other providers and not given adequate care to what they require personally. It is my job as a social worker in the health care field to be a voice, supporter, motivator, and educator to patients with the aid of a cohesive palliative care team, with an end goal of putting the patient first.
	- As a practitioner, I will be an advocate for palliative care. I believe it should be mentioned to all cancer patients just as education at first. This will open the door for patients to learn more and know that if they choose to be comfortable, instead of pursuing curative treatment, palliative care will provide multiple areas of support for them. Palliative care opens a door to allow a patient to achieve their goals, live as they want, spend time with family, and do legacy work before they can no longer.
	- Working with the team at Hospice and the client/family made me feel good. I felt like I was doing a good deed and working more for Jesus than myself. This type of work is fulfilling to me. It makes me feel positive and useful. Although end of life isn’t a happy time, I feel good in knowing that I’m doing something amazing that all people can’t do.
	- This experience impacted the way that I approached all patient interactions that followed it in a positive way and I have gained more confidence in communication with patients. I am able to express appropriate emotions without feeling exposed, wrong, or out of line. In my future practice, I will take into consideration the state of the patient and their personal team’s understanding and processing of what is going on.
	- I think working with cancer patients and their families is very humbling and has taught me a great deal of compassion. I am now softer when it comes to approaching those who are sick and the family members as well. I have learned [from Mrs. B’s husband of 50 years] the true meaning of being there for someone and actually showing it.
	- This reaffirmed my desire to be an oncology social worker. I do not have a fear of death or dealing with dying people. I feel that the elderly have so much to offer and I enjoy listening to their life stories.

**Table 6:** Quotes related to the subtheme of the centrality of communication.

Subtheme	Quotations
*Centrality of Communication*	
**Good Communication**	- I personally learned…the worth of open-ended questions.
	- I also realized how helpful it could be to accept the silence of the patient and wait until the point she was ready to open up.
	- I have learned the importance of developing rapport with patients and their loved ones.
	- The hospice team has an amazing communication process.
	- Communication, as in many cases, was a key factor in this case.
	- The oncology team provides quality care in that they do attempt to maintain communication with one another.
	- I learned how family sessions are conducted, what influences the decision for palliative care, and the importance of communication in an interdisciplinary team.
**Poor Communication**	- The interdisciplinary team may have learned more about the patient and her family if everyone discussed what they observed and did for Mrs. B.
	- I believe that we could have better served T and his family if the doctors sat in rounds with us.
	- The communication among everyone could have been improved because Mr. S needed his transplant very soon and he and his family still didn’t know the process of the transplant.
	- I felt that it would benefit the patient if she and her husband could address several of their concerns/needs at once rather than talking with different team members at separate times.

**Table 7:** Quotes related to the subtheme of importance of social support.

Subtheme	Quotations
**Importance of Social Support**	- As a future healthcare provider, I must acknowledge that not all people will have the same support system as Mr. J. I must always remember that I need to work especially hard to address the social support needs of the patient and family.
	- This experience impacted my future practice as a healthcare provider by showing me it takes a village when trying to improve the patient’s quality of life.
	- I have learned that it is important to talk to the patient’s family in order to discover the support of the patient.
	- Her support system consisted solely of her husband. This patient needs an expanded positive support system.
	- The individuals without family need more from a social worker and that is an area where we need to constantly improve our skills and competencies.
	- When Mr. S reported there weren’t many support people I was very sympathetic towards him because I couldn't imagine not having anyone to help me through this process.

**Table 8:** Quotes related to the subtheme of appreciation of family as the unit of care.

Subtheme	Quotations
**Family as the Unit of Care**	- Palliative care does not just improve the care of the patient; it improves the care of the family.
	- As a student social worker, working with the W family was meaningful for me because it gave me an insight on the family functioning side of cancer. When a person has cancer, it not only affects them but also their loved one and I was able to see how it affects a husband who is also a caretaker to his wife. Professionally, I was able to assess how stress and depression can occur in both the person with and without cancer and how as a caretaker it can be just as high as their loved one who is ill. Mr. W is suffering right along with his wife and it is evident because he also asks to speak with my supervisor for supportive counseling. He states that he likes speaking with my supervisor because she has been able to be a support to him and his wife since the beginning of her cancer journey.
	- The team did well to involve all members of the team, rather than solely relying on the doctor to be the deciding factor in D’s treatment. All members took time to discuss her case with each other and the family to determine the next steps and how it affected the family.
	- Working in social work with the end of life population, I have found that palliative care is an essential tool and resource that the entire family dynamic can benefit from. The family was a perfect candidate for palliative care, coming to the conclusion of palliative care they seemed relieved with the decision they made. I fully believe being able to provide peace of mind to families who are heartbroken and trying to balance the emotional and the rational side of making end of life decisions is humbling and beneficial to the families.
	- I feel that effective palliative care can be a gift to both the patient and the family.

**Table 9:** Quotes related to the subtheme of countertransference.

Subtheme	Quotations
**Countertransference**	- I have had several family members who have fought cancer, both successfully and unsuccessfully. Through this experience, I have observed several of the needs and forms of care that cancer patients require that I have not considered when attending to my own family members. It was hard not to picture my family when looking at this vulnerable patient.
	- Several times throughout this process, I thought back to my father’s own diagnosis and treatments and the way he was treated by his doctors and nurses. Although his prognosis is not considered end of life, it gives me hope and assurance that that type of care would be available if necessary.
	- This moment brought back memories from the experience that I had when my aunt had cancer. One second, we were getting a call telling us she was no longer in remission, to a second call telling us she had weeks, to a third call telling us she had days. In my supervisor’s office, many emotions overcame me and I felt that because I had gone through something similar, I could understand her confusion and fear. I had not previously been aware of this patient or his diagnosis. In this moment, I realized that it is normal to feel emotion and to be touched by patient experiences that do not directly affect me.

**Table 10:** Quotes related to the subtheme of emotional reactions.

Subtheme	Quotations
**Emotional Reactions**	- I was personally touched by the way the patient was dealing with the situation.
	- For me, this case was very sad. It was my first case where I was able to meet a patient and follow them until their passing.
	- It was touching to see how life crises can bring families and especially couples even closer.
	- The experience of meeting Ms. F. was very touching to me because it allowed me a look into her world.
	- Some points during my rotation were absolutely heartbreaking, but for the most part it was an incredibly rich experience.
	- I was inspired by the love and caring support of Mr. J. and his future caregivers. It seems all anyone ever hears about is the lack of caring and compassion of families, of how we just drop our so-called loved ones in nursing homes or other care facilities and leave them to fend for themselves; I did not witness this. I always find it difficult to see the swiftness in which one’s health can turn, but today I was rewarded with the opportunity to see how a family takes compassionate action to care for their loved one.
	- The situation for me personally is frightening.
	- It touched my heart to see so many patients trying.
	- I was personally touched by the dedication of the caregiver.
	- It made me really sad…
	- I was rewarded with the opportunity to see how a family takes compassionate action to care for their loved one.
	- I think working with cancer families is very humbling and it has taught me a great deal of compassion.
	- Seeing S stay strong for her family and willing to fight her cancer as long as possible in whatever way necessary, made me feel stronger.
	- There were also a few heartbreaking things that happened that I could not believe.
	- I was impressed with the strengths of the patient and family.

**Table 11:** Quotes related to the subtheme of conflict between personal and patient/family values.

Subtheme	Quotations
**Conflict Between Personal and Patient/Family Values**	- Overall, this experience provided me with great insight about difficult cases and the personal issues that can arise in myself from working in palliative care.
	- I had hoped [he] would allow his grandchildren to see him and felt it was selfish of him to not allow that. Upon reflection, I considered that he might have thought it was selfish of him to want to see them bearing in mind his condition. This act of reflection reminded me of my biases and that it was good to keep them from interfering with my work.
	- It was a little difficult for me to talk about religion with T because I do not share the same faith as him and I didn’t want to offend or upset him by saying so. I feel comfortable telling people that I’m a Hindu, but I have learned from the past that very religious Christians usually feel a duty to teach me about Jesus. When T would talk about God and Jesus, I politely agreed and affirmed his feelings about the matter. However, I did not engage in that topic too much because I was afraid of offending him. The chaplain had been contacted to see T and his family, but he liked to share his religious views with all the medical and hospital staff that saw him. It did not offend me at all, but I was just worried that I would offend him. Perhaps this is something that I need to explore much deeper because I’m sure it will arise again, especially if I work with people in a palliative care or hospice care setting.

## Supplementary Materials

The following are available online at http://www.mdpi.com/2308-3417/3/1/6/s1, critical reflective writing experience assignment description.
