# Supplementary material for: “Sacred Work”: Reflections on the Professional and Personal Impact of an Interdisciplinary Palliative Oncology Clinical Experience by Social Work Learners
Source: Geriatrics (Basel). 2018 Feb 3;3(1):6. doi: 10.3390/geriatrics3010006 (PMC6371177; doi:10.3390/geriatrics3010006)
Supplement: Supplementary file 1 [file geriatrics-03-00006-s001.pdf]

UNIVERSITY OF LOUISVILLE

Interdisciplinary
Curriculum for
Oncology
Palliative Care
Education

Critical Reflection Writing and Group Experience

For this assignment, you are to focus on one patient that you observed during your palliative care clinical experience. Address the areas described below.

1) In 1-2 paragraphs, summarize the clinical scenario in language that non-clinical readers could understand.
    - **Briefly** describe the patient and his/her situation, diagnosis and prognosis, brief history of the disease, and how/why the patient entered palliative-focused care.
    - Describe the predominant palliative care needs of this patient and his/her family. Think holistically about their medical, psychosocial and spiritual needs.
    - Briefly describe how well these needs are currently being met. What team members are involved and what interventions are occurring? How do you judge the progress being made towards the goals for this patient's care?

2) Now – put on your critical thinking cap. Address the following critical review questions.
    - How could the patient's/family's care be enhanced?
    - Was there an adequate interdisciplinary assessment and care plan?
    - Describe the communication among the parties involved (team communication: with each other, the patient and family and other providers)
    - Discuss how this interdisciplinary and patient/family oriented setting supported quality palliative care. If it didn't, how did it fall short?

3) Lastly but most importantly, reflect personally on what you learned from your involvement with this patient. This should be the longest portion of your paper.
    - How did the experience touch you personally? Professionally?
    - How did the experience impact your future practice as a healthcare provider?
    - Describe observations that had a positive or negative impact on you.
    - How has your perception of palliative care changed based on the experience?
